# A Questionnaire Study on the Use of Complementary and Alternative Veterinary Medicine for Dogs in Sweden

**DOI:** 10.3390/vetsci8120331

**Published:** 2021-12-15

**Authors:** Lisa Sohlberg, Anna Bergh, Susanna Sternberg-Lewerin

**Affiliations:** 1 Trestads Djurklinik, 46273 Vänersborg, Sweden; sohlberglisa@gmail.com; 2 Department of Clinical Sciences, Swedish University of Agricultural Sciences, 75007 Uppsala, Sweden; anna.bergh@slu.se; 3 Department of Biomedical Sciences & Veterinary Public Health, Swedish University of Agricultural Sciences, 75007 Uppsala, Sweden

**Keywords:** CAVM, CAM, treatment, rehabilitation, therapist

## Abstract

Complementary and alternative veterinary medicine (CAVM) includes treatment methods that are not part of established veterinary medicine and where there is not as yet sufficient scientific documentation of the proposed effects. The CAVM use in Swedish dogs is largely unknown, and the aim of this study was to explore this use. Electronic questionnaires were distributed to dog owners, small animal veterinary practitioners, and CAVM therapists. A total of 253 dog owners responded. Except for massage, stretching, and laser therapy, more than 75% of the respondents stated that they did not use CAVM for their dogs. Of the 216 responding veterinarians, 39% did not use CAVM. CAVM use was more common among respondents with <10 years of work experience as compared to those with >10 years of work experience (*p* < 0.05). Almost half of the 124 responding CAVM therapists treated dogs; the most frequently used methods included massage, stretching, and acupressure. A majority of all respondents found the current Swedish regulation of CAVM insufficient. Although CAVM use in Swedish dogs appears to be uncommon, there is a need for scientific assessment of CAVM in dogs as regards safety and effectiveness for different indications.

## 1. Introduction

The interest in complementary and alternative veterinary medicine (CAVM) has increased in the last decades [1,2]. CAVM includes a broad spectrum of treatment methods that are not part of established veterinary medicine and where there is limited scientific documentation of the proposed effects [3,4,5,6]. Some methods may be regarded as ineffective, whereas others are close to being considered conventional veterinary medicine. In addition, some methods may have a demonstrated effect for some indications in humans [7] but lack scientific documentation in animals. In the “AVMA (American Veterinary Medical Association) guidelines for complementary and alternative medicine”, CAVM is defined as “…a heterogeneous group of preventive, diagnostic, and therapeutic philosophies and practices. The theoretical bases and techniques of CAVM may diverge from veterinary medicine routinely taught in veterinary medical schools or may differ from current scientific knowledge, or both.” [8]. However, as the definition of CAVM differs between countries and changes over time, the exact demarcation between CAVM and classic veterinary medicine is not fixed [9,10].

CAVM is not directly regulated in Sweden but falls under the legislation on animal welfare and operations within animal healthcare and veterinary medicine [11]. Hence, CAVM methods and CAVM therapists are not assessed or evaluated by any authority or quality assurance organisation. According to the animal welfare regulation [12], animal owners are obliged to consult a veterinarian if the need arises and might be regarded as in violation of this law if a CAVM therapist is contacted instead of a veterinarian, if the animal is in need of veterinary care.

Most Swedish dogs are insured [13], and, after an injury, the insurance companies covers the costs of some CAVM methods for rehabilitation, such as swimming, stretching, massage, and treadmill. A prerequisite for payment is, however, that a veterinarian has written a referral for the treatment. Swedish veterinarians are legally required to work in an evidence-based way and use scientific knowledge in their practice, and this requirement presents a challenge, as most CAVM methods have not been evaluated for safety and efficacy for specific indications in animals.

Animal owners increasingly demand CAVM from veterinarians, but there is not sufficient scientific support for including CAVM in the veterinary curriculum [14]. However, many veterinary faculties offer courses in CAVM, mostly acupuncture, physiotherapy, herbal medicine, and chiropractic [1]. The main argument for this is that veterinarians need to be prepared for questions about CAVM, but any CAVM course in the veterinary curriculum must be evidence-based [1]. It has been suggested that elective courses on CAVM are a good option [15]. Animal owners appear to have a more positive attitude towards CAVM than veterinarians [14], similarly to human medicine, where medical students become increasingly critical towards CAM during their years of medical education [16,17].

In human medicine, complementary and alternative medicine (CAM) is used by many patients in combination with, or besides, conventional medicine [18]. Patients do not always inform their doctor about their CAM use [19,20], and this can become a problem in offering optimal medical care [18]. Similarly, a study showed that horse owners in New Zealand use CAVM without telling their veterinarian [21]. 

For the past 20 years, 17–71% of the Swedish population have used CAM, mainly massage, different CAM products, acupuncture, chiropractic, and naprapathy [7]. In a US study, 65% of 254 owners of cats and dogs with cancer used CAVM [22], but the CAVM use among Swedish dog owners is largely unknown.

The objective of this study was to describe the use of and attitudes to CAVM among Swedish dog owners, Swedish small animal practitioners, and Swedish CAVM therapists.

## 2. Materials and Methods

The study was performed as an MSc project at the Swedish University of Agricultural Sciences [23], in parallel to a similar project on horse owners and equine veterinary practitioners [24]. Electronic questionnaires were constructed and distributed to dog owners, small animal veterinary practitioners, and CAVM therapists as described in Gilberg et al. [24], and the therapist questionnaire was the same as in that study. Briefly, the dog owner questionnaires were distributed via social media, and the questionnaires for veterinarians and therapists were distributed via professional organisations. The translated questionnaires are provided as Supplementary Material Table S1. In addition to demographic data for the respondents, the questions included frequency, type, and reasons for CAVM use, as well as money spent on CAVM (dog owners) and attitudes to regulation of CAVM.

The questionnaires were pilot tested on a few veterinary students and/or dog owners (*n* = 10) and adapted according to comments from these persons. The pilot test did not include any data collection, and the test persons were only asked if the questionnaire was user-friendly.

Data were analysed by the Chi-square test, after extraction into Microsoft^®^ Excel (Microsoft Co., Redmond, WA, USA) spreadsheets.

## 3. Results

### 3.1. Dog Owners

The dog owner questionnaire attracted a total of 253 people, of which 105 responded to all questions. The response rate varied for each question. The majority of the 253 respondents (92%) were female, and about half (51%) lived in rural areas. Most were between 18 and 65 years of age, and 45% lived in the south, 39% in the middle, and 16% in the north of the country. Most (64%) had received post-high school education, while 29% had high school level education (*n* = 253). Nearly half (48%) kept their dogs as companions, while 35% stated dog shows as the main purpose (*n* = 248), and most (67%) had more than 5 years’ experience as dog owners.

There was no significant difference between the respondents who used their dogs for companionship or shows and those who used them for more physically exerting purposes (agility, hunting, etc.) as regards to responses to the question: “Has you dog ever been lame or experienced other locomotor problems?”. “Yes” was replied by 145 of 248 (58%), and 67 (46%) of these described the problems as recurring. Of the respondents who replied “Yes” to the questions about locomotor problems, 77% had consulted a veterinarian, while 87% of those with recurring problems had taken their dog to a veterinarian. The CAVM methods most used by the respondents whose dogs had not been seen by a veterinarian (*n* = 33) included massage (42%), stretching (39%), ceramic fabric (30%), and laser therapy (21%). When suspecting lameness in their dog, 84% of 224 respondents stated that they first consult a veterinarian, 14% that they consult a CAVM therapist, and 2% chose “other”. The corresponding proportions for suspicion of back pain (*n* = 218) were 86%, 12%, and 2%. The respondents who chose “other” wrote, e.g., “feed additives” or “it depends on the cause”.

Stretching, massage, and laser therapy were the most commonly used methods. Table 1 illustrates the frequency of use of the different methods for therapeutic reasons (i.e., rehabilitation after injury) or prevention (i.e., on healthy dogs). 

Half (50%) of the respondents (*n* = 133) stated that the CAVM treatment had helped their dog, while 27% were unsure and 9% replied that the treatment had not helped. The only method where the negative responses were not clearly in a minority was homeopathy, where almost half of the respondents using this method stated that it had not helped.

Further, one respondent (*n* = 86) had observed a negative side effect of the CAVM treatment but did not specify the side effect.

Out of the 55 respondents who had contacted a CAVM therapist, 10 (18%) had been referred by a veterinarian, 27 (49%) had been recommended by a friend, one had seen an advertisement, and 17 chose the option “other”. The clarifications for “other” included responses indicating that the contact had been established since long ago or the respondent was a therapist themselves. There was no significant association between type of method and reason for contact; a friend more commonly recommended all methods used than based on referral by a veterinarian.

The respondents were also asked to indicate what method they believed were most effective for different types of injuries. Overall, the respondents believed that CAVM was most effective for treating lameness and back pain, where 18% replied that they did not think any CAVM method was effective, while 56–79% gave the same response for other disorders (gastrointestinal problems, skin problems, problems related to airways, weight loss, oral cavity problems, and behavioural problems). The apparently most trusted methods were (in order of preference): massage, ceramic fabric (reflecting body heat), stretching, laser therapy, chiropractic, and acupuncture, where >25% (*n* = 118) believed they would work for lameness, back pain, or both.

**Figure 1:**
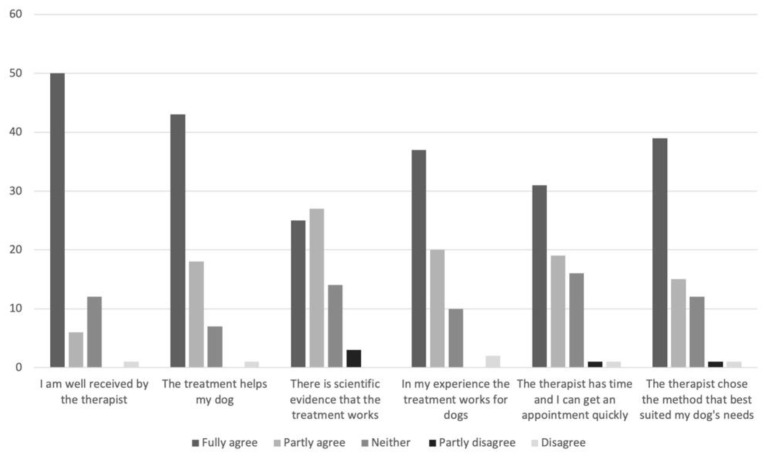
Responses by Swedish dog owners (*n* = 69) to the question “If your dog has been treated with complementary and alternative veterinary medicine, why did you choose that therapist and/or method?”.

**Figure 2:**
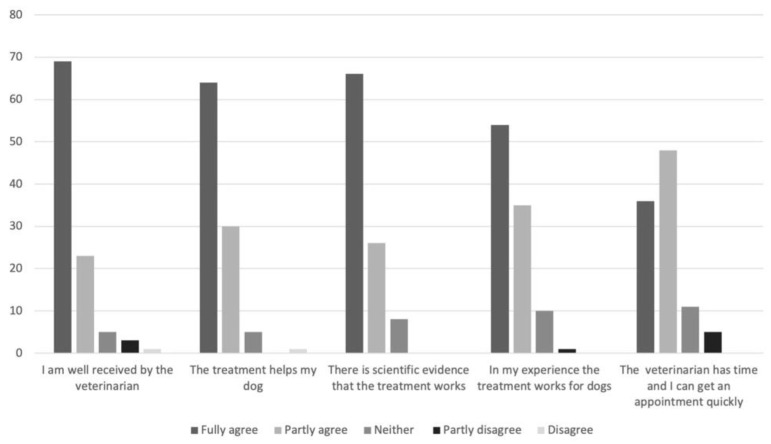
Responses by Swedish dog owners (*n* = 100) to the question “If you have consulted a veterinarian when your dog was sick or injured, why?”.

In Figure 1, the respondents’ agreements to different statements on why they had chosen to contact a CAVM therapist are shown, and the corresponding responses for reasons to contact a veterinarian are shown in Figure 2.

A space for free text responses was also provided; these included statements about using CAVM as a complement to veterinary care, wanting to reduce the use of pharmaceuticals, shorter waiting time for an appointment with the therapist, and the therapist having better knowledge about locomotion and the entire body. The corresponding free text responses to why a veterinarian was consulted included reasons such as veterinarians having a long education and a high level of competence, that some ailments must be treated medically, and that veterinary medicine is based on evidence.

The responses to the questions about how much money the dog owners had spent on preventive CAVM treatments in the last year revealed that 63% had spent up to 1000 Swedish Krona (SEK), 23% had spent between 1000 and 5000 SEK, and 14% had spent >5000 SEK (*n* = 101). For treatments of injuries, 77% had spent up to 1000 SEK, 16% between 1000 and 5000 SEK, and 7% had spent >5000 SEK (*n* = 101) on CAVM.

The corresponding expenditure on products for CAVM, for prevention or treatment were: 59% had spent up to 1000 SEK, 36% had spent between 1000 and 5000 SEK, and 5% had spent >5000 SEK (*n* = 103).

About half (51%) of the respondents did not know if their insurance policy would cover treatments by non-veterinarians, while 29% knew this to be the case, and 20% knew this was not the case (*n* = 105). Most (57%) responded that they had not thought much about this issue, 20% believed it was important, and 23% did not consider it important. There was no association between money spent on CAVM and knowledge of insurance policy covering CAVM.

### 3.2. Veterinarians

Among the members of the Swedish Veterinary Association (just over 3200 veterinarians, of which at least 600 mainly work in small animal medicine), 216 small animal practitioners responded to the questionnaire, of which 162 completed it. The response rate varied between the questions. Of the 216 respondents, the majority (84%) were female, 47% lived in the south of Sweden, 44% in the middle, and 9% in the north. More than half (69%) worked in urban areas. The age distribution was fairly even, ranging between 24 years and >65 years, with slightly fewer in the oldest group. Most (79%) were educated in Sweden, 20% were educated in other European countries, and <1% were trained outside Europe. The veterinary education had not included CAVM for 78% of the 2016 respondents, while 7% said CAVM had been included in their training (13 of these were educated in Sweden and three in other European countries) and 14% did not remember. Fifteen respondents (7%) had postgraduate training in CAVM and five in acupuncture, while other responses included chiropractic, laser therapy, feed additives, physiotherapy, and homeopathy. 

More than a third (39%) did not use any CAVM method in their work. There was no difference between female and male respondents as regards CAVM use. However, 72% of the respondents with less than 10 years of work experience used CAVM while the corresponding figure for respondents with >10 years of work experience was 55% (*p* < 0.05). Of those that did use CAVM, most used stretching, massage, laser therapy, and water treadmill. Except for laser and water treadmill, the same methods were most common among those who used CAVM on their own dogs (50 of the 216 respondents stated that they did not own a dog). Ceramic fabrics were also used for both patients and privately owned dogs, see Table 2.

**Figure 3:**
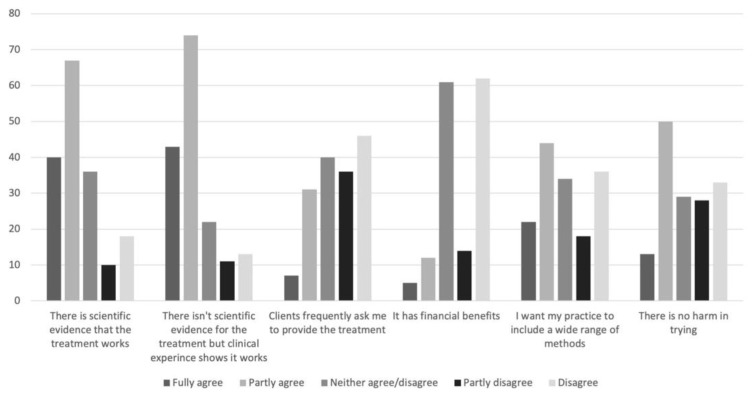
Reasons for using complementary and alternative veterinary medicine (CAVM), as stated by Swedish small animal veterinarians responding to a questionnaire (*n* = 171, 163, 160, 154, 154, 153 in order shown, i.e., decreasing for each new question).

In Figure 3, the reasons for using or recommending CAVM, as stated by the veterinarians, are shown. For each of these questions, there was a reduction in the number of respondents. This could be interpreted either as weariness or objections to the phrasing of the questions from some of them.

Table 3 shows the different indication for the CAVM methods used, as stated by those respondents who used them. As in previous questions, the most common methods were massage, water treadmill, stretching, and laser. However, most respondents stated that they do not use the method themselves, as they “refer to the rehab department”. Among the explanations for “other”, statements about referring or consulting a physiotherapist, rehabilitation exercises, and swimming were mentioned. Other responses included statements that each injury must be individually assessed and properly diagnosed.

Most respondents rarely see patients who have been referred by a CAVM therapist, 39% stated that this never happened, 39% estimated it to occur <5 times per year, and 37 replied that it happens >6 times/year. On the other hand, 56% (*n* = 168) of the respondents stated that they refer their patients to CAVM therapists, while 44% stated that they have never done this. The vast majority (98%) of the explanatory responses include statements about referral to rehabilitation/a physiotherapist.

Most (63%, *n* = 147) replied “Yes” to the question about whether they know the level of education of the CAVM therapist that they refer to. Free text responses included statements about “only refer to people I know” and “only refer to those with qualified education”. When asked if they follow up the CAVM treatment they refer to, 32% (*n* = 134) responded that they book a follow-up visit, 19% that they do not follow up on the treatment, and 17% that they stay in touch with the CAVM therapist. Clarifying responses included statements about only referring “in-house” to the rehab department, and “follow-up via telephone if the therapist does not work in the same clinic”.

### 3.3. CAVM Therapists

Of the 124 therapists who responded to the questionnaire, 107 completed it. As this questionnaire is the same as in Gilberg et al. [24], only data directly relevant for this study are presented here, while other detailed results can be found in the referred paper. Most of the respondents (69%) worked with multiple species, and about half (48%, *n* = 116) treated dogs. The most common methods included massage, stretching, and acupressure (see Table 4). The methods not used by the therapists reporting that they treated dogs have been excluded from the table. However, it is not known for what animal species each method was used.

Treatment of animals constituted the main source of income for 37% of 123 respondents. The majority (98%, *n* = 123) were educated in Sweden, but 16% also had some training outside Sweden. In the free text box, half of the respondents stated that they had attended an animal massage school. 

Most (97%, *n* = 118) stated that animal owners contact them directly, but many also obtain referrals from veterinarians and other CAVM therapists (multiple responses were possible). Most (72%) stated that they collaborate with a veterinarian, and 25% responded that they do not. 

**Figure 4:**
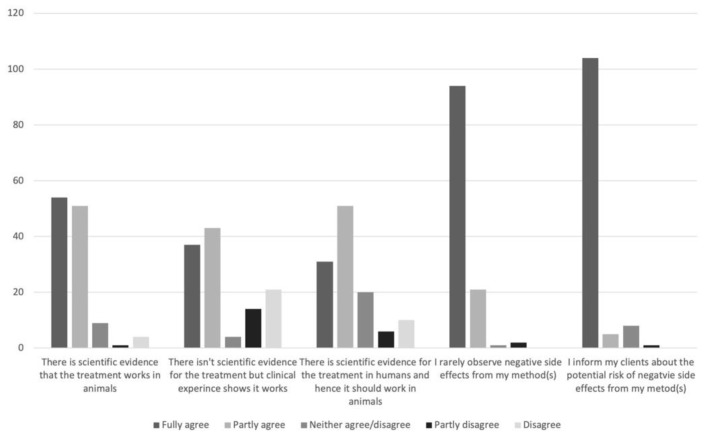
Perceptions of methods used, as reported by Swedish complementary and alternative veterinary medicine (CAVM) therapists responding to a questionnaire.

Figure 4 illustrates the CAVM therapists’ perceptions of the methods they use.

### 3.4. Legal Regulation of CAVM

The final question in all questionnaires was if and how the use of CAVM in animals should be regulated with the aim to improve animal welfare and avoid mistreatments. Several options could be chosen in this question, and free text was also allowed. The responses are shown in Table 5.

In the free text box, responses from veterinarians included statements that there is no evidence for CAVM and hence it should not be used, that it is impossible for animal owners to assess CAVM methods and therapists, and that there is a lack of evidence for many conventional methods and hence CAVM should not be defined as “alternative”. Free text responses from therapists included a call for more collaboration between veterinarians and therapists and that they keep records.

## 4. Discussion

Compared to Swedish horse owners [24], Swedish dog owners seem to rarely use CAVM for their dogs, but the same methods (massage and stretching) appear to be the most common. This was mostly a reflection of the methods preferred by CAVM therapists, although the ranking of methods such as acupressure and laser therapy differed somewhat between dog owners and therapists.

Research into this area is needed, with the aim to protect animal welfare and increase the knowledge in the area. Although the number of responses to each questionnaire was low, the geographical distribution can be seen as representative and, as this is the first study on CAVM use in dogs, at least in Sweden, the results may serve as a basis for further studies. Selection bias cannot be ruled out, as it is likely that respondents interested in CAVM were more eager to participate. The gender bias among the respondents could be linked to men being less interested in CAVM or a gender imbalance among the participants in the social media groups where the questionnaire was advertised. Previously reported associations between CAVM use and gender, age, and income [25,26,27] could not be verified in this study. 

The CAVM methods listed in the questionnaires were selected based on previous studies and available materials on CAVM [28,29,30]. As there are many definitions for CAVM, methods that are included in conventional human medicine have been listed as CAVM, based on their inconclusive scientific documentation of clinical efficacy for specified species. The questionnaire was pilot tested and adapted accordingly, but some questions may still have been misunderstood by the respondents. The reasons for those respondents who chose not to complete the questionnaire can only be speculated.

A majority of the participating dog owners first contacted a veterinarian in case of lameness or back pain in their dog, but some stated that a CAVM therapist was the first contact. This could increase the risk of an aggravated problem if a medical diagnosis is not obtained in the initial phase. Most CAVM consultations appeared to be based on recommendations from a friend, indicating that more information to dog owners about the evidence basis of different methods is needed. Most dog owners may hesitate to discuss CAVM, as it is hard to know whether to raise the issue or wait for the veterinarian to bring it up. The same applies to the veterinarian, who may not want to risk any loss of confidence and subsequent lack of treatment compliance [7,9]. More dog owners agreed fully to the statement about scientific evidence for veterinary medicine than for the corresponding statement about CAVM, indicating that they are aware of the lack of evidence for CAVM but may choose it anyway. Some free text comments about wanting to reduce the use of pharmaceuticals, quicker appointments with CAVM therapists, and holistic thinking among CAVM therapists may indicate a mindset among the dog owners who choose CAVM. The veterinary education presumably provides veterinarians with a holistic knowledge, as the education covers multiple aspects of a sick or injured animal, but their clients may not perceive this. In human medicine, patients with back pain are reported to be more prone to use CAVM due to a wish to avoid prescription medicines with negative side effects [31]. A similar thinking may be the reason for dog owners’ choice of CAVM, despite the awareness of the limited scientific documentation. In addition, most CAVM methods have a shorter withdrawal period before competitions than pharmaceuticals such as NSAID (non-steroid anti-inflammatory drugs), a fact that might lead owners of competing dogs to prefer CAVM.

The large interest in CAVM among pet owners means that veterinarians need to be prepared for questions about CAVM [1]. Regardless of the veterinarians’ attitude towards CAVM, a factual summary of the pros and cons should be provided to those clients who raise the subject. They may choose CAVM anyway, but it is preferable that this is communicated to the veterinarian, as it can be assumed that lack of such communication is a problem in veterinary medicine as well as in human medicine [18].

The collaborations between veterinarians and CAVM therapist appear to vary: more than a third of the veterinarians stated that they never received patients on referral from CAVM therapists, and almost half that they have never referred to a CAVM therapist. However, some free text responses included statements such as “I refer in-house to the rehab department”. There was no difference among these respondents as regards working in a rural area or a city, but it could be assumed that animal hospitals/larger clinics would be more likely to have a rehab unit than smaller clinics. Some other free text comments included statements about using ceramic fabric rugs because of the good fit, not because of the health benefit claims of the producer, and “I wouldn’t use CAVM even if I owned a dog”. Half of the therapists stated that they receive referrals form veterinarians, which is in line with the responses of the veterinarians. On the other hand, most therapists (72%) claimed to refer to a veterinarian, if necessary, while 25% did not have any collaboration with a veterinarian.

There was no clear association between the level of education of the CAVM therapists and the method they used. This was a little unexpected as some methods do not require any formal education while, e.g., human chiropractic requires a university education. On the other hand, in animal therapy, there are few protected titles, as well as hardly any specific academic qualification. Nevertheless, it may not matter that the therapist has a long education in a method that has no effect in animals. Many therapists responded that there is good evidence for the method they use, which might be a misconception based on either a lack of knowledge or the referral to scientific documentation in humans. This further emphasises that research on the clinical effectiveness of CAVM for specific indications in animals is highly needed. 

The regulation of CAVM is a controversial issue that differs from CAM. People can choose for themselves to try out different treatments and take responsibility for their perception of the treatment and its effects. Animals can, like young children, not describe their perceptions, and are dependent on their owners/parents. Because of this, CAM treatment of children below the age of eight years is prohibited in Swedish legislation [32]. Similarly, based on the survey results, a regulation of CAVM could be considered, as very few respondents in any category felt that the legal regulation of CAVM needs no change.

Many dog owners and veterinarians wanted a requirement for veterinary consultation before CAVM use, but few of the therapists. The majority of all respondents agreed with the statement that the therapist should be obliged to refer to a veterinarian when needed, which is already implied in the animal welfare regulation. Not surprisingly, few veterinarians thought that they should be obliged to refer to a CAVM therapist when needed. Most respondents also thought a requirement for record systems for CAVM therapists should be introduced. Some therapists already keep records but not all. Patient record systems would facilitate follow-up and treatment evaluation and should be a basic requirement in any care of a sick or injured animal. More than half of the respondents thought basic training in animal medicine should be part of all CAVM education. Besides more knowledge of the efficacy of each method, better knowledge of anatomy, physiology, and pathology among CAVM therapists would most likely benefit animal welfare and reduce the risk of incorrect treatments. More therapists and dog owners than veterinarians wanted protected titles for CAVM therapists. Protected titles would probably benefit all actors, as they would enable assessment of the qualifications of the therapists. However, the requirement for evidence of effective use in animals remains, and a requirement to first see a veterinarian would still be needed for a proper diagnosis. 

## 5. Conclusions

Based on our results, CAVM use appears to be uncommon in Swedish dogs, and there seems to be little collaboration between veterinarians and CAVM therapists. Those that use CAVM mainly use stretching, massage, and laser therapy. Protected titles would enable assessment of the qualifications of the CAVM therapists. However, evidence of effectiveness of the method in animals is still required, to protect animal welfare. 

## Figures and Tables

**Table 1:** Frequency of use of complementary and alternative veterinary medicine methods used for either therapy or prevention, as stated by Swedish dog owners responding to a questionnaire (*n* ranging from 194 to 188).

Method	>4 Times/Year	Rarely	Not at All
Acupuncture	7 (4%) *	14 (7%)	173 (89%)
Ceramic fabric	20 (10%)	11 (6%)	163 (84%)
Chiropractic	18 (9%)	24 (13%)	148 (78%)
Cooling mud	2 (1%)	6 (3%)	180 (96%)
Craniosacral therapy	3 (2%)	0 (0%)	187 (98%)
Electrotherapy/TENS/NMES	13 (7%)	11 (6%)	170 (88%)
Healing	6 (3%)	5 (3%)	182 (94%)
Homeopathy	3 (2%)	8 (4%)	181 (94%)
Hair analysis	1 (0.5%)	2 (1%)	187 (98%)
Iris diagnostics	1 (0.5%)	1 (0.5%)	188 (99%)
Kinesiology	3 (2%)	4 (2%)	183 (97%)
Laser therapy	33 (17%)	29 (15%)	128 (67%)
LED light therapy	4 (2%)	5 (3%)	180 (95%)
Magnetic therapy	2 (1%)	3 (2%)	184 (97%)
Massage	71 (38%)	40 (21%)	78 (41%)
Naprapathy	5 (3%)	8 (4%)	176 (93%)
Osteopathy	3 (2%)	2 (1%)	184 (97%)
Ointment	7 (4%)	13 (7%)	168 (89%)
Stretching	73 (39%)	30 (16%)	86 (45%)
Therapeutic ultrasound	5 (3%)	7 (4%)	176 (94%)

* Proportions indicate distribution of responses for each method. TENS = transcutaneous electrical nerve stimulation; NMES = neuromuscular electrical stimulation.

**Table 2:** Methods used by Swedish small animal veterinarians responding to a questionnaire on complementary and alternative veterinary medicine (CAVM) (*n* = 216, multiple choices possible).

Method	Use for Patients	Use for Own Dog ^a^
Acupuncture	16 (7%)	6 (3%)
Ceramic fabric	48 (22%)	16 (7%)
Chiropractic	3 (1%)	2 (1%)
Cooling mud	5 (2%)	0
Electrotherapy/TENS/NMES	6 (3%)	2 (1%)
Homeopathy	1 (0.5%)	1 (0.5%)
Kinesiology	0	0
Laser therapy	44 (20%)	16 (7%)
LED light therapy	0	0
Magnetic therapy	0	0
Massage	61 (28%)	28 (13%)
Naprapathy	1 (0.5%)	1 (0.5%)
Ointment	6 (3%)	1 (0.5%)
Osteopathy	1 (0.5%)	0
Stretching	49 (23%)	24 (11%)
Therapeutic ultrasound	3 (1%)	1 (0.5%)
Water treadmill	77 (36%)	19 (9%)
Other ^b^	33 (15%)	4 (2%)
Don’t use any CAVM	85 (39%)	104 (48%)

^a^ 50 respondents did not own a dog. ^b^ Free text includes physiotherapy, swimming, vibration therapy, and molecular medicine, plus explanations about sometimes referring to a CAVM therapist.

**Table 3:** Complementary and alternative veterinary medicine (CAVM) methods used or recommended for different indications by Swedish small animal veterinarians responding to a questionnaire. (*n* = 216, multiple choices possible).

Method	Tendon Injury	Muscle Injury	Skeletal Injury	Ligament Injury	Arthritis	Neural Injury	Back Pain
Acupuncture	2	7	3	2	3	8	15
Chiropractic	1	9	5	2	5	4	8
Electrotherapy	2	11	1	3	4	12	9
Laser therapy	12	10	2	12	1	6	25
LED light	1	1	0	1	0	0	0
Magnetic therapy	1	1	0	2	1	1	1
Massage	4	34	8	6	7	13	62
Naprapathy	0	2	1	1	2	1	2
Osteopathy	0	3	2	2	2	2	0
Stretching	3	31	4	6	6	8	23
Ultrasound	2	2	0	4	0	0	31
Water treadmill	7	12	7	8	8	8	56
None of the above	56	19	42	31	35	31	19
Other	17	13	10	14	12	10	15

**Table 4:** Complementary and alternative veterinary medicine (CAVM) methods used on animals by Swedish therapists responding to a questionnaire (*n* = 116, multiple choices possible).

Method		Method	
Acupressure	70 (56%)	Massage	106 (85%)
Acupuncture	45 (36%)	Mobilisation	34 (27%)
Aromatherapy	1 (1%)	Moxibustion	12 (10%)
Aquatherapy/hydrotherapy	1 (1%)	Myofascial release	29 (23%)
Chiropractic	15 (12%)	Naprapathy	8 (6%)
Colloidal silver	3 (2%)	Osteopathy	21 (17%)
Craniosacral therapy	4 (3%)	Reflexology	2 (2%)
Crystal therapy	2 (2%)	Shockwave therapy	4 (3%)
Distance healing	3 (2%)	Sound therapy	2 (2%)
Electrotherapy	13 (10%)	Stretching	72 (58%)
Healing	11 (9%)	Swimming	2 (2%)
Herbal medicine	11 (9%)	Therapeutic exercise	29 (23%)
Hirudotherapy	1 (1%)	Traditional Chinese medicine	25 (20%)
Homeopathy	7 (6%)	Trigger point therapy	44 (35%)
Infrasound	5 (4%)	Ultrasound therapy	4 (3%)
Kinesiology	13 (10%)	Vibration therapy	8 (6%)
Laser therapy	56 (45%)	Vitamin/mineral therapy	3 (2%)
LED light therapy	11 (9%)	Water therapy	7 (6%)
Light therapy	3 (2%)	Water treadmill	10 (8%)
Ointment	19 (15%)	Zone therapy	3 (2%)
Magnetic therapy	7 (6%)	Other	28 (23%)
Manipulation	23 (19%)		

**Table 5:** The responses of Swedish dog owners (*n* = 106), small animal veterinary practitioners (*n* = 167) and complementary and alternative veterinary medicine (CAVM) therapists (*n* = 116) to a question about regulation of CAVM in animals.

	Dog Owners	Veterinarians	Therapists
The current situation is fine, no changes needed	1 (1%)	4 (2%)	8 (7%)
A veterinary consultation should be required before CAVM treatment	53 (50%)	75 (45%)	5 (4%)
The CAVM therapist should be obliged to refer to a veterinarian if needed	75 (71%)	116 (69%)	90 (78%)
The veterinarian should be required to refer to a CAVM therapist if needed.	23 (22%)	7 (4%)	39 (34%)
Requirements on record systems for CAVM therapists should be introduced.	59 (56%)	127 (76%)	70 (60%)
Any CAVM education should include basic training in animal medicine	55 (52%)	101 (60%)	67 (58%)
Protected professional titles for CAVM therapists should be introduced	61 (58%)	69 (41%)	69 (59%)

## Data Availability

Data in coded format can be obtained from the last author.

## Supplementary Materials

The following are available online at https://www.mdpi.com/article/10.3390/vetsci8120331/s1, Table S1: Translated questionnaires.
