# Supplementary material for: A Questionnaire Study on the Use of Complementary and Alternative Veterinary Medicine for Dogs in Sweden
Source: Vet Sci. 2021 Dec 15;8(12):331. doi: 10.3390/vetsci8120331 (PMC8704802; doi:10.3390/vetsci8120331)
Supplement: Supplementary file 1 [file vetsci-08-00331-s001.zip › vetsci-1480504-supplementary.pdf]

Table S1. A questionnaire study on the use of complementary and alternative veterinary medicine for dogs in Sweden L. Sohlberg, A. Bergh, S. Sternberg-Lewerin *Veterinary Sciences*

Dog owner questionnaire

1. What is you gender?

- ☐ Female
- ☐ Male
- ☐ Non-binary
- ☐ Do not want to say

2. How old are you?

- ☐ <18 years
- ☐ 18-23 years
- ☐ 24-35 years
- ☐ 36-50 years
- ☐ 51-64 years
- ☐ >65 years

3. Where do you live?

- ☐ Norrland (North Sweden)
- ☐ Svealand (Middle Sweden)
- ☐ Götaland (South Sweden)

4. Do you live in a town or a rural area?

- ☐ Town
- ☐ Rural area

5. What is your educational background?

- ☐ Grundskola (elementary, 9 years)
- ☐ Gymnasieskola (high school, 3 years)
- ☐ Eftergymnasial (university or other, after 12 years)
- ☐ Do not want to say

6. For how long have you owned a dog (or had full responsibility for a dog)?

- ☐ 0-5 years
- ☐ 6-10 years
- ☐ 11-20 years
- ☐ >21 years

7. In what discipline(s) is your dog active?

- ☐ Agility
- ☐ Working dogs

- ☐ Obedience
- ☐ Rally obedience
- ☐ Show
- ☐ Hunting
- ☐ My dog is mainly a companion
- ☐ Other

8. Has your dog evet been lame or had other locomotor system problems? (the locomotor system includes legs, muscles, joints, tendons and back)

- ☐ Yes
- ☐ No

9. If yes in the previous question, has the dog had recurrent problems?

- ☐ Yes
- ☐ No

10. Has your dog been examined by a veterinarian in relation to a locomotor system injury?

- ☐ Yes
- ☐ No

11a. When you suspect lameness in your dog, who do you contact first for treatment? (CAVM = complementary and alternative veterinary medical treatment methods)

- ☐ Veterinarian
- ☐ CAVM-therapist
- ☐ Other ________________________________

11b. When you suspect back pain in your dog, who do you contact first for treatment?

- ☐ Veterinarian
- ☐ CAVM-therapist
- ☐ Other ________________________________

12a. Is your dog treated with any of the following and in that case how often?
<u>After</u> disease/injury

	>2 times/ month	1-2 times/ month	Every 2nd month	Every 3rd/4th month	More rarely	Do not use
Acupuncture	☐	☐	☐	☐	☐	☐
Electrotherapy, e.g. TENS	☐	☐	☐	☐	☐	☐
Healing	☐	☐	☐	☐	☐	☐
Homeopathy	☐	☐	☐	☐	☐	☐
Hair analysis	☐	☐	☐	☐	☐	☐

Iris diagnostics	☐	☐	☐	☐	☐	☐
Kinesiology	☐	☐	☐	☐	☐	☐
Chiropractic	☐	☐	☐	☐	☐	☐
Craniosacral therapy	☐	☐	☐	☐	☐	☐
Laser therapy	☐	☐	☐	☐	☐	☐
LED light therapy	☐	☐	☐	☐	☐	☐
Magnetic field therapy	☐	☐	☐	☐	☐	☐
Massage	☐	☐	☐	☐	☐	☐
Naprapathy	☐	☐	☐	☐	☐	☐
Osteopathy	☐	☐	☐	☐	☐	☐
Stretching	☐	☐	☐	☐	☐	☐
Therapeutic ultrasound	☐	☐	☐	☐	☐	☐
Ointment	☐	☐	☐	☐	☐	☐
Cooling mud	☐	☐	☐	☐	☐	☐
Ceramic fabric	☐	☐	☐	☐	☐	☐

Do you use any other CAVM-method <u>after</u> disease/injury that was not listed above? If yes, please state what method and how often used.

____
____
____

Has the treatment helped your dog?

☐ No
☐ Do not know
☐ Yes
☐ Free text ___________________________________

12b. Is your dog treated with any of the following and in that case how often?
<u>To prevent</u> disease/injury

	>2 times/ month	1-2 times/ month	Every 2nd month	Every 3rd/4th month	More rarely	Do not use
Acupuncture	☐	☐	☐	☐	☐	☐
Electrotherapy, e.g. TENS	☐	☐	☐	☐	☐	☐
Healing	☐	☐	☐	☐	☐	☐
Homeopathy	☐	☐	☐	☐	☐	☐
Hair analysis	☐	☐	☐	☐	☐	☐
Iris diagnostics	☐	☐	☐	☐	☐	☐

Kinesiology	☐	☐	☐	☐	☐	☐
Chiropractic	☐	☐	☐	☐	☐	☐
Craniosacral therapy	☐	☐	☐	☐	☐	☐
Laser therapy	☐	☐	☐	☐	☐	☐
LED light therapy	☐	☐	☐	☐	☐	☐
Magnetic field therapy	☐	☐	☐	☐	☐	☐
Massage	☐	☐	☐	☐	☐	☐
Naprapathy	☐	☐	☐	☐	☐	☐
Osteopathy	☐	☐	☐	☐	☐	☐
Stretching	☐	☐	☐	☐	☐	☐
Therapeutic ultrasound	☐	☐	☐	☐	☐	☐
Ointment	☐	☐	☐	☐	☐	☐
Cooling mud	☐	☐	☐	☐	☐	☐
Ceramic fabric	☐	☐	☐	☐	☐	☐

Do you use any other CAVM-method <u>to prevent</u> disease/injury that was not listed above? If yes, please state what method and how often used.
____
____
____

13. Has your dog experienced any negative side effects from CAVM treatments used?

☐ Yes

☐ No

☐ Comment __________________________________

14. If your dog has been treated with CAVM, what made you contact the CAVM therapist?

☐ Recommended by a friend

☐ Referral by a veterinarian

☐ Advertisement

☐ Have not contacted any therapist

☐ Other __________________________________

15. What method do you think has a good effect for treatment of the following injuries? (Multiple alternatives can be chosen)

<u>Lameness</u>

☐ Acupuncture

☐ Electrotherapy/muscle stimulation/TENS NMES

☐ Healing

- ☐ Homeopathy
- ☐ Hair analysis
- ☐ Iris diagnostics
- ☐ Kinesiology
- ☐ Chiropractic
- ☐ Craniosacral therapy
- ☐ Laser therapy
- ☐ LED light therapy
- ☐ Magnetic/magnetic field therapy
- ☐ Massage
- ☐ Naprapathy
- ☐ Osteopathy
- ☐ Stretching
- ☐ Therapeutic ultrasound
- ☐ Ointment
- ☐ Cooling mud
- ☐ Ceramic fabric
- ☐ None of the above
- ☐ Other ________________________________

Back problems

- ☐ Acupuncture
- ☐ Electrotherapy/muscle stimulation/TENS NMES
- ☐ Healing
- ☐ Homeopathy
- ☐ Hair analysis
- ☐ Iris diagnostics
- ☐ Kinesiology
- ☐ Chiropractic
- ☐ Craniosacral therapy
- ☐ Laser therapy
- ☐ LED light therapy
- ☐ Magnetic/magnetic field therapy
- ☐ Massage
- ☐ Naprapathy
- ☐ Osteopathy
- ☐ Stretching

- ☐ Therapeutic ultrasound
- ☐ Ointment
- ☐ Cooling mud
- ☐ Ceramic fabric
- ☐ None of the above
- ☐ Other ________________________________

Gastrointestinal problems

- ☐ Acupuncture
- ☐ Electrotherapy/muscle stimulation/TENS NMES
- ☐ Healing
- ☐ Homeopathy
- ☐ Hair analysis
- ☐ Iris diagnostics
- ☐ Kinesiology
- ☐ Chiropractic
- ☐ Craniosacral therapy
- ☐ Laser therapy
- ☐ LED light therapy
- ☐ Magnetic/magnetic field therapy
- ☐ Massage
- ☐ Naprapathy
- ☐ Osteopathy
- ☐ Stretching
- ☐ Therapeutic ultrasound
- ☐ Ointment
- ☐ Cooling mud
- ☐ Ceramic fabric
- ☐ None of the above
- ☐ Other ________________________________

Skin problems

- ☐ Acupuncture
- ☐ Electrotherapy/muscle stimulation/TENS NMES
- ☐ Healing
- ☐ Homeopathy
- ☐ Hair analysis

- ☐ Iris diagnostics
- ☐ Kinesiology
- ☐ Chiropractic
- ☐ Craniosacral therapy
- ☐ Laser therapy
- ☐ LED light therapy
- ☐ Magnetic/magnetic field therapy
- ☐ Massage
- ☐ Naprapathy
- ☐ Osteopathy
- ☐ Stretching
- ☐ Therapeutic ultrasound
- ☐ Ointment
- ☐ Cooling mud
- ☐ Ceramic fabric
- ☐ None of the above
- ☐ Other ________________________________

Respiratory problems

- ☐ Acupuncture
- ☐ Electrotherapy/muscle stimulation/TENS NMES
- ☐ Healing
- ☐ Homeopathy
- ☐ Hair analysis
- ☐ Iris diagnostics
- ☐ Kinesiology
- ☐ Chiropractic
- ☐ Craniosacral therapy
- ☐ Laser therapy
- ☐ LED light therapy
- ☐ Magnetic/magnetic field therapy
- ☐ Massage
- ☐ Naprapathy
- ☐ Osteopathy
- ☐ Stretching
- ☐ Therapeutic ultrasound
- ☐ Ointment

- ☐ Cooling mud
- ☐ Ceramic fabric
- ☐ None of the above
- ☐ Other ________________________________

Weight loss

- ☐ Acupuncture
- ☐ Electrotherapy/muscle stimulation/TENS NMES
- ☐ Healing
- ☐ Homeopathy
- ☐ Hair analysis
- ☐ Iris diagnostics
- ☐ Kinesiology
- ☐ Chiropractic
- ☐ Craniosacral therapy
- ☐ Laser therapy
- ☐ LED light therapy
- ☐ Magnetic/magnetic field therapy
- ☐ Massage
- ☐ Naprapathy
- ☐ Osteopathy
- ☐ Stretching
- ☐ Therapeutic ultrasound
- ☐ Ointment
- ☐ Cooling mud
- ☐ Ceramic fabric
- ☐ None of the above
- ☐ Other ________________________________

Oral cavity problems

- ☐ Acupuncture
- ☐ Electrotherapy/muscle stimulation/TENS NMES
- ☐ Healing
- ☐ Homeopathy
- ☐ Hair analysis
- ☐ Iris diagnostics
- ☐ Kinesiology

- ☐ Chiropractic
- ☐ Craniosacral therapy
- ☐ Laser therapy
- ☐ LED light therapy
- ☐ Magnetic/magnetic field therapy
- ☐ Massage
- ☐ Naprapathy
- ☐ Osteopathy
- ☐ Stretching
- ☐ Therapeutic ultrasound
- ☐ Ointment
- ☐ Cooling mud
- ☐ Ceramic fabric
- ☐ None of the above
- ☐ Other ________________________________

Behavioural problems

- ☐ Acupuncture
- ☐ Electrotherapy/muscle stimulation/TENS NMES
- ☐ Healing
- ☐ Homeopathy
- ☐ Hair analysis
- ☐ Iris diagnostics
- ☐ Kinesiology
- ☐ Chiropractic
- ☐ Craniosacral therapy
- ☐ Laser therapy
- ☐ LED light therapy
- ☐ Magnetic/magnetic field therapy
- ☐ Massage
- ☐ Naprapathy
- ☐ Osteopathy
- ☐ Stretching
- ☐ Therapeutic ultrasound
- ☐ Ointment
- ☐ Cooling mud
- ☐ Ceramic fabric

☐ None of the above

☐ Other ________________________________

16. If your dog has been treated by any of the previously mentioned methods, for what reason did you chose that method or therapist? Select for each statement how well it matches you and your dog.

	Fully agree	Partly agree	Neither	Partly disagree	Disagree
I am well received by the therapist	☐	☐	☐	☐	☐
The treatment helps my dog	☐	☐	☐	☐	☐
There is scientific evidence that the treatment works	☐	☐	☐	☐	☐
In my experience the treatment works for dogs	☐	☐	☐	☐	☐
The therapist has time and I can get an appointment quickly	☐	☐	☐	☐	☐
The therapist chose the method that best suited my dog's needs	☐	☐	☐	☐	☐

17. If your dog has been by a veterinarian for disease/injury, for what reason did you chose to contact the veterinarian? Select for each statement how well it matches you and your dog.

	Fully agree	Partly agree	Neither	Partly disagree	Disagree
I am well received by the veterinarian	☐	☐	☐	☐	☐
The treatment helps my dog	☐	☐	☐	☐	☐
There is scientific evidence that	☐	☐	☐	☐	☐

the treatment works					
In my experience the treatment works for dogs	☐	☐	☐	☐	☐
The veterinarian has time and I can get an appointment quickly	☐	☐	☐	☐	☐

Any other reasons for choosing CAVM treatment?
____
____
____

Any other reasons for consulting a veterinarian?
____
____
____

18. How much have you spent on <u>preventive</u> treatments (those listed in previous questions) for your dog in the last year?

☐ 0-1 000 SEK
☐ 1 001-5 000 SEK
☐ 5 001-10 000 SEK
☐ >10 000 SEK

19. How much have you spent on <u>products</u> for <u>treatment/prevention</u> for your dog's health in the last year?

☐ 0-1 000 SEK
☐ 1 001-5 000 SEK
☐ 5 001-10 000 SEK
☐ >10 000 SEK

20. How much have you spent on CAVM treatments (those listed in previous questions) for <u>treatment of disease or injury</u> in your dog in the last year?

☐ 0-1 000 kr
☐ 1 001-5 000
☐ 5 001-10 000
☐ mer än 10 000

21. Does your insurance company pay for treatments by non-veterinarians, e.g. those listed in previous questions?

- ☐ Yes
- ☐ Yes, on veterinary referral
- ☐ No
- ☐ Don't know

22. Is it important when you choose insurance company that the previously mentioned methods are compensated?

- ☐ Yes
- ☐ No
- ☐ Haven't thought about it

23. In what way do you think CAVM treatment of animals should be regulated to improve animal welfare and avoid potential incorrect treatments? (Multiple alternatives can be selected)

- ☐ Things are fine, nothing needs changing
- ☐ A veterinary consultation should be mandatory before CAVM treatment, to avoid incorrect treatment
- ☐ The therapist should be required to refer to a veterinarian if needed
- ☐ The veterinarian should be required to refer to a CAVM therapist if needed
- ☐ A requirement for record keeping should be introduced, to increase patient safety and facilitate follow-up
- ☐ Basic knowledge of animal medicine should be included in all CAVM education
- ☐ Protected professional titles should be introduced for CAVM therapists, so that animal owners know who has what qualifications
- ☐ Own suggestion ________________________________

Thank you for responding to the questionnaire! If you have any additional comments, please feel free to write them here

____
____

Questionnaire to small animal veterinarians

1. What is you gender?

- ☐ Female
- ☐ Male
- ☐ Non-binary
- ☐ Do not want to say

2. How old are you?

- ☐ <18 years
- ☐ 18-23 years

- ☐ 24-35 years
- ☐ 36-50 years
- ☐ 51-64 years
- ☐ >65 years

3. Where do you live?

- ☐ Norrland (North Sweden)
- ☐ Svealand (Middle Sweden)
- ☐ Götaland (South Sweden)

4. Do you work in a town or a rural area?

- ☐ Town
- ☐ Rural area

5. For how long have you been a veterinary practitioner?

- ☐ <5 years
- ☐ 6-10 years
- ☐ 11-20 years
- ☐ 21-30 years
- ☐ >31 years

6. In which country did you receive your veterinary education?

- ☐ Sweden
- ☐ Other Nordic country
- ☐ Other European country
- ☐ Outside Europe

7. Did your veterinary curriculum include CAVM (complementary and alternative veterinary medicine)?

- ☐ Yes
- ☐ No
- ☐ Cannot remember

8. Have you received postgraduate training in CAVM?

- ☐ No
- ☐ Yes, in what subject: ___________________________________

9. How many canine patients do you see per week with locomotor problems?

- ☐ <10
- ☐ 11-29
- ☐ >30

10. What are your canine patients commonly used for? (Multiple alternatives can be selected)

- ☐ Agility
- ☐ Working dogs
- ☐ Obedience
- ☐ Rally obedience
- ☐ Show
- ☐ Hunting
- ☐ Mostly companions
- ☐ Other

11. Do you use any CAVM method professionally? (Multiple alternatives can be selected)

- ☐ Acupuncture
- ☐ Electrotherapy (e.g. TENS)
- ☐ Homeopathy
- ☐ Kinesiology
- ☐ Chiropractic
- ☐ Laser therapy
- ☐ LED light therapy
- ☐ Magnetic/magnetic field therapy
- ☐ Massage
- ☐ Naprapathy
- ☐ Osteopathy
- ☐ Stretching
- ☐ Therapeutic ultrasound
- ☐ Water treadmill
- ☐ Ointment
- ☐ Cooling mud
- ☐ Ceramic fabric
- ☐ No
- ☐ Other_________________________________

12. Do you use any CAVM method privately (in your own dog)? (Multiple alternatives can be selected)

- ☐ Acupuncture
- ☐ Electrotherapy (e.g. TENS)
- ☐ Homeopathy
- ☐ Kinesiology

- ☐ Chiropractic
- ☐ Laser therapy
- ☐ LED light therapy
- ☐ Magnetic/magnetic field therapy
- ☐ Massage
- ☐ Naprapathy
- ☐ Osteopathy
- ☐ Stretching
- ☐ Therapeutic ultrasound
- ☐ Water treadmill
- ☐ Ointment
- ☐ Cooling mud
- ☐ Ceramic fabric
- ☐ No
- ☐ Other________________________________

13. If you use or recommend any CAVM method, for what reason do you choose that method? Select for each statement how well it agrees with your opinion.

	Fully agree	Partly agree	Neither	Partly disagree	Disagree
There is scientific evidence that the treatment works	☐	☐	☐	☐	☐
The method lacks scientific evidence but there is clinical experience	☐	☐	☐	☐	☐
I am often asked by clients to provide the treatment	☐	☐	☐	☐	☐
It has economic benefits	☐	☐	☐	☐	☐
I want my practice to include a broad selection of methods	☐	☐	☐	☐	☐

It doesn't hurt to try	☐	☐	☐	☐	☐

14. For what indication(s) do you use/recommend any of the following methods? (Multiple alternatives can be selected)

Tendon injury

☐ Acupuncture
☐ Electrotherapy e.g. TENS
☐ Chiropractic
☐ Laser therapy
☐ LED light therapy
☐ Magnetic/magnetic field therapy
☐ Massage
☐ Naprapathy
☐ Osteopathy
☐ Stretching
☐ Therapeutic ultrasound
☐ Water treadmill
☐ None of the above
☐ Other ________________________________

Muscle injury

☐ Acupuncture
☐ Electrotherapy e.g. TENS
☐ Chiropractic
☐ Laser therapy
☐ LED light therapy
☐ Magnetic/magnetic field therapy
☐ Massage
☐ Naprapathy
☐ Osteopathy
☐ Stretching
☐ Therapeutic ultrasound
☐ Water treadmill
☐ None of the above
☐ Other ________________________________

Skeletal injury

- ☐ Acupuncture
- ☐ Electrotherapy e.g. TENS
- ☐ Chiropractic
- ☐ Laser therapy
- ☐ LED light therapy
- ☐ Magnetic/magnetic field therapy
- ☐ Massage
- ☐ Naprapathy
- ☐ Osteopathy
- ☐ Stretching
- ☐ Therapeutic ultrasound
- ☐ Water treadmill
- ☐ None of the above
- ☐ Other ________________________________

Ligament injury

- ☐ Acupuncture
- ☐ Electrotherapy e.g. TENS
- ☐ Chiropractic
- ☐ Laser therapy
- ☐ LED light therapy
- ☐ Magnetic/magnetic field therapy
- ☐ Massage
- ☐ Naprapathy
- ☐ Osteopathy
- ☐ Stretching
- ☐ Therapeutic ultrasound
- ☐ Water treadmill
- ☐ None of the above
- ☐ Other ________________________________

Arthritis

- ☐ Acupuncture
- ☐ Electrotherapy e.g. TENS
- ☐ Chiropractic
- ☐ Laser therapy
- ☐ LED light therapy

- ☐ Magnetic/magnetic field therapy
- ☐ Massage
- ☐ Naprapathy
- ☐ Osteopathy
- ☐ Stretching
- ☐ Therapeutic ultrasound
- ☐ Water treadmill
- ☐ None of the above
- ☐ Other ________________________________

Neural injury

- ☐ Acupuncture
- ☐ Electrotherapy e.g. TENS
- ☐ Chiropractic
- ☐ Laser therapy
- ☐ LED light therapy
- ☐ Magnetic/magnetic field therapy
- ☐ Massage
- ☐ Naprapathy
- ☐ Osteopathy
- ☐ Stretching
- ☐ Therapeutic ultrasound
- ☐ Water treadmill
- ☐ None of the above
- ☐ Other ________________________________

Back problem

- ☐ Acupuncture
- ☐ Electrotherapy e.g. TENS
- ☐ Chiropractic
- ☐ Laser therapy
- ☐ LED light therapy
- ☐ Magnetic/magnetic field therapy
- ☐ Massage
- ☐ Naprapathy
- ☐ Osteopathy
- ☐ Stretching

☐ Therapeutic ultrasound

☐ Water treadmill

☐ None of the above

☐ Other ________________________________

15. Do you receive clients that have been recommended by a CAVM therapist to consult a veterinarian?

☐ Never happens

☐ <5 times/year

☐ 6-20 times/year

☐ 21-40 times/year

☐ >40 times/year

16. Do you refer your patients to a CAVM therapist?

☐ Never done

☐ Yes – how often and to what type of therapist? ________________________________

17. Do you know the level of education to the therapist(s) you refer to?

☐ No

☐ Yes ________________________________

18. If you refer to CAVM therapists, how do you follow up the treatment?

☐ Book an appointment with me or a colleague

☐ Contact the therapist

☐ Don't follow up the treatment

☐ Other ________________________________

19. In what way do you think CAVM treatment of animals should be regulated to improve animal welfare and avoid potential incorrect treatments? (Multiple alternatives can be selected)

☐ Things are fine, nothing needs changing

☐ A veterinary consultation should be mandatory before CAVM treatment, to avoid incorrect treatment

☐ The therapist should be required to refer to a veterinarian if needed

☐ The veterinarian should be required to refer to a CAVM therapist if needed

☐ A requirement for record keeping should be introduced, to increase patient safety and facilitate follow-up

☐ Basic knowledge of animal medicine should be included in all CAVM education

- ☐ Protected professional titles should be introduced for CAVM therapists, so that animal owners know who has what qualifications
- ☐ Own suggestion ________________________________

Thank you for responding to the questionnaire! If you have any additional comments, please feel free to write them here
____
____

CAVM therapist questionnaire

1. What is you gender?
- ☐ Female
- ☐ Male
- ☐ Non-binary
- ☐ Do not want to say

2. How old are you?
- ☐ <18 years
- ☐ 18-23 years
- ☐ 24-35 years
- ☐ 36-50 years
- ☐ 51-64 years
- ☐ >65 years

3. Where do you live?
- ☐ Norrland (North Sweden)
- ☐ Svealand (Middle Sweden)
- ☐ Götaland (South Sweden)

4. Do you work in a town or a rural area?
- ☐ Town
- ☐ Rural area

5. Which patients do you treat?

	Often (several times/week)	Sometimes	Rarely	Never
People	☐	☐	☐	☐
Horses	☐	☐	☐	☐
Dogs	☐	☐	☐	☐
Cats	☐	☐	☐	☐

Other animals	☐	☐	☐	☐

6. What CAVM method(s) do you use for treatment of animals? (CAVM = complementary and alternative veterinary medicine). More than one alternative can be selected.

☐ Acupressure
☐ Acupuncture
☐ Anthroposofic medicine
☐ Aroma therapy
☐ Auriculotherapy
☐ Aquatherapy/hydrotherapy
☐ Bio Harmony
☐ Bioresonance therapy
☐ Chiropractic
☐ Colloidal silver
☐ Craniosacral therapy
☐ Crystal therapy
☐ Distance healing
☐ DMSO (dimethyl sulfoxide)
☐ Electrotherapy (NMES, TENS)
☐ Gold injection
☐ Healing
☐ Herbal medicine
☐ Homeopathy
☐ Water therapy
☐ Infrasound
☐ Ion therapy
☐ Iontoforesis
☐ Iris diagnostics
☐ Kinesiology
☐ Laser therapy
☐ LED light therapy
☐ Hirudotherapy (leeches)
☐ Light therapy
☐ Ointment
☐ Magnetic therapy
☐ Manipulation
☐ Massage

- ☐ Mesotherapy
- ☐ Mobilisation
- ☐ Moxibustion
- ☐ Mud treatment
- ☐ Myofascial release
- ☐ Naprapathy
- ☐ Neural therapy
- ☐ Osteopathy
- ☐ Reflexology
- ☐ Shock wave treatment
- ☐ Sound treatment
- ☐ Stretching
- ☐ Swimming
- ☐ Therapeutic exercise
- ☐ Traditional Chinese medicine
- ☐ Trigger point therapy
- ☐ Therapeutic ultrasound
- ☐ Vacuum therapy
- ☐ Vibration therapy
- ☐ Vitamin- and mineral therapy
- ☐ Water treadmill
- ☐ Zone therapy
- ☐ Other ________________________________

7. Is treatment of animals your main source of income?

- ☐ Yes
- ☐ No

8. Have you received training in the method(s) you use? (More than one alternative can be selected)

- ☐ Separate courses provided by a private actor
- ☐ Educational programme provided by a private actor
- ☐ Separate university-level courses
- ☐ University-level programme
- ☐ No formal training, have taught myself
- ☐ No formal training, have been taught by a friend
- ☐ Other

9. Have you finished your training or are you studying now?

- ☐ Currently studying
- ☐ Finished training

10. Where did you receive your education?

- ☐ Sweden
- ☐ Abroad
- ☐ If you like, please describe: ________________________________

11. For how long have you been practising the methods mentioned above? If you use more than one method, respond for the method you have practised for the longest time.

- ☐ <5 years
- ☐ 6-10 years
- ☐ 11-30 years
- ☐ >30 years

12. How do you recruit you patients?

- ☐ Via veterinarians
- ☐ Via other CAVM therapists
- ☐ Directly via the animal owner

13. What is the nature of your collaboration with veterinarians?

- ☐ No collaboration
- ☐ Refer/recommend my patients to a veterinarian when needed
- ☐ Get referrals/recommendations from veterinarian(s) when needed

14. How do you follow up the treatment of a patient that you have referred to a veterinarian?

- ☐ Book an appointment for follow up with me or a non-veterinary colleague
- ☐ Contact with the veterinarian
- ☐ Do not follow up treatment
- ☐ Other ________________________________

15. Please select for each of the following statements how well it matches your opinion

	Fully agree	Partly agree	Neither	Partly disagree	Disagree
There is scientific evidence that my method(s) work for animals	☐	☐	☐	☐	☐
There isn’t scientific evidence, but experience shows that that my method(s) work for animals	☐	☐	☐	☐	☐

There is scientific evidence that my method(s) work for humans, and hence they should work for animals	☐	☐	☐	☐	☐
I rarely observe any negative side effects from my method(s)	☐	☐	☐	☐	☐
I inform the client about the potential risk of negative side effects from my method(s)	☐	☐	☐	☐	☐

16. In what way do you think CAVM treatment of animals should be regulated to improve animal welfare and avoid potential incorrect treatments? (Multiple alternatives can be selected)

☐ Things are fine, nothing needs changing
☐ A veterinary consultation should be mandatory before CAVM treatment, to avoid incorrect treatment
☐ The therapist should be required to refer to a veterinarian if needed
☐ The veterinarian should be required to refer to a CAVM therapist if needed
☐ A requirement for record keeping should be introduced, to increase patient safety and facilitate follow-up
☐ Basic knowledge of animal medicine should be included in all CAVM education
☐ Protected professional titles should be introduced for CAVM therapists, so that animal owners know who has what qualifications
☐ Own suggestion ________________________________

Thank you for responding to the questionnaire! If you have any additional comments, please feel free to write them here

____
____
